# Investigating the role of flight phase and task difficulty on low-time pilot performance, gaze dynamics and subjective situation awareness during simulated flight

**DOI:** 10.16910/jemr.17.1.6

**Published:** 2024-06-17

**Authors:** Naila Ayala, Suzanne Kearns, Elizabeth Irving, Shi Cao, Ewa Niechwiej-Szwedo

**Affiliations:** University of Waterloo, Ontario, Canada

**Keywords:** Eye movement, gaze entropy, areas of interest, visual scanning, aviation training, flight simulation

## Abstract

Gaze behaviour has been used as a proxy for information processing capabilities that underlie complex skill performance in real-world domains such as aviation. These processes are highly influenced by task requirements, expertise and can provide insight into situation awareness (SA). Little research has been done to examine the extent to which gaze behaviour, task performance and SA are impacted by various task manipulations within the confines of early-stage skill development. Accordingly, the current study aimed to understand the impact of task difficulty on landing performance, gaze behaviour and SA across different phases of flight. Twenty-four low-time (<300 hours) pilots completed simulated landing scenarios under visual flight rules conditions. Traditional gaze metrics, entropybased metrics, and blink rate provided meaningful insight about the extent to which information processing is modulated by flight phase and task difficulty. The results also suggested that gaze behavior changes compensated for increased task demands and minimized the impact on task performance. Dynamic gaze analyses were shown to be a robust measure of task difficulty and pilot flight hours. Recommendations for the effective implementation of gaze behaviour metrics and their utility in examining information processing changes are discussed.

## Introduction

Most of the information regarding flight controls and operation is gathered and processed by the human visual system. Since modern cockpits present a complex human-machine interface with multiple competing stimuli, pilots must be able to optimally scan and process critical pieces of information for the safe and successful operation of an aircraft. As such, eye-tracking research in the aeronautical domain has largely focused on the development and implementation of eye-tracking metrics with the objective of understanding what constitutes superior pilot monitoring of aircraft controls in expert pilots (26; 40; 64). Accordingly, several studies have already identified changes in traditional and advanced gaze (i.e., coordinated head and eye movements) metrics in expert pilots that vary with task difficulty, which are associated with differences in pilot performance and situation awareness (SA) (12; 26; 42; for review see 40 and 64). However, what remains is a specific gap in understanding how these measures are impacted within the confines of early-stage pilot skill development. Therefore, the aim of the current study was to understand the impact of task difficulty on flight performance, gaze behaviour and SA across various phases of flight in low-time (i.e., <300 hours) pilots using a high-fidelity simulator environment.

Eye-tracking offers insight into skill performance by providing both behavioural and physiological outputs that can be used to provide insight regarding pilot performance, as well as the underlying cognitive processes (15; 29; 32; 42; 62). For instance, it has been well established that high performing pilots spend more time selectively allocating their visual attention towards objects that are relevant to the task goals, while ignoring other task irrelevant areas (1; 18; 26; 27; 30; 43; 54). Specifically, optical splay angle and runway length-width ratio have be referenced as helpful runway visual cues that improve landing performance (8; 30; 35). These apparent gaze biases are not only highly task dependent but demonstrate how high performing pilots efficiently and effectively scan their environment to sample all necessary information required to successfully plan and complete a specific task. What is important to note is that skill performance in itself may be very similar between any two pilots, or two groups of pilots. However, the way in which information is processed during a given task may differ despite their comparable performance capabilities. Eye tracking can help reveal these differences through the examination of other basic and dynamic gaze metrics that provide further insight into how efficiently individuals process information and could reveal the differing levels of task demands associated with a particular scenario. For example, previous work has shown that more efficient information processing is associated with reduced fixation duration (3; 12; 26; 40; 50; 52; 64), increased fixation frequencies (12; 25; 26; 28; 40; 64), as well as a greater propensity to fixate more task-relevant areas of interest (AOIs) - greater fixation dispersion (i.e., Stationary Gaze Entropy: SGE) - in a more variable/flexible pattern; as made apparent with greater fixation sequence complexity (i.e., Gaze Transition Entropy: GTE) (6; 5; 4; 26; 28; 40; 50). Several studies have also demonstrated how reduced blink rate is a reliable proxy for increased task difficulty as well as cognitive load (26; 40). The application and exploration of these basic and dynamic gaze metrics remain important areas of inquiry with respect to pilot training and skill mastery because even if performance is optimal in an error-free state of flight, there may be serious ramifications to flight performance and safety should an emergency/error arise; especially, if such an event is associated with an overload in the pilots’ information processing capabilities.

Studies examining gaze behaviour in pilots across various stages of flight demonstrated distinct gaze behaviour changes as task demands changed (5; 7; Comstock, 1995; 17; 33; Lijing et al., 2014; 57). To our knowledge, only two studies have specifically investigated the impact of differing task demands on flight performance and gaze behaviour in low-time pilots (5; 17). Ayala and colleagues (5) assessed gaze behaviour in 18 low-time pilots (flight hour range: 0-240, mean= 64 hours, SD= 91) as they completed simulated landing scenarios of varying difficulty (i.e., easy: no wind, high visibility; difficult: high winds, high visibility) programmed in a desktop computer Microsoft Flight Simulator game environment (2020, Asobo Studio, France). Results showed that an increase in task difficulty was associated with a longer dwell time toward the runway, along with a reduction in fixation sequence dispersion and complexity. Moreover, prolonged fixation on a singular AOI (i.e., front window)- also known as cognitive tunneling (9; 22; 56)- became evident, demonstrating a reduction in pilot monitoring of internal cockpit gauges as a result of increased task difficulty. Therefore, it was concluded that gaze in low-time pilots became less complex and more focal; thus, making the scanning and processing of information more targeted toward task relevant AOIs when task difficulty increased. Similarly, Dehais and colleagues (17) assessed gaze behaviour in 7 low-time pilots (flight hour range: 80-250 hours), as they completed two traffic patterns and basic flight maneuvers in a real aircraft. Results demonstrated significant changes in gaze dwell time patterns as a function of task demands (i.e., subgoals) imposed by different stages of flight (i.e., take-off, downwind, final approach). Although this study focused exclusively on characterizing the dwell time patterns across AOIs during different stages of flight, it was instrumental to showing how eye-tracking could be used for training purposes (i.e., informing pilot monitoring strategies) as well as highlighting the importance of accounting for stages of flight in gaze analytics.

The present study sought to expand on previous work in three important respects. First, the current study specifically focused on pilots with their Private Pilots’ License (PPL) or Commercial Pilots’ License (CPL). Earlier work demonstrated that there is a significant learning curve that occurs within the ab-initio stage of skill development that is associated with more variability around the performance and gaze measures of interest that may go unexplained and impact our conclusions (5). Furthermore, the use of high-fidelity flight simulators has been shown to immediately diminish the performance of ab-initio pilots who are unfamiliar with the immersive cockpit environment (38). As such, using strict recruitment criteria to define the cohort was seen as a necessary step to reduce the heterogeneity across participants, and specifically understand how gaze, flight performance and SA interact as a function of task difficulty across flight phases in the cluster of pilots who are licensed to fly a single pilot aircraft. Second, the use of a high-fidelity flight simulator in the current study provides more data related to aircraft control and landing performance throughout the duration of the trial. Indeed, one of the limitations with using Microsoft Flight Simulator in previous work was that there were no continuous measures of aircraft control that could be examined in line with continuous measures of gaze behaviour. This limited the ability to examine the ongoing dynamic interactions between gaze and aircraft control during all phases of landing (i.e., downwind, base, final approach) in the simulated scenario. Lastly, the current investigation included an assessment of situation awareness in the form of a SA questionnaire (Situational Awareness Rating Technique: SART; 53) to assess the relationship between gaze behaviour and SA in low-time pilots. Although other questionnaires for SA exist (i.e., Situation Awareness Global Assessment Technique: SAGAT) (20), they require the pausing of the flying scenario to ask probing questions. This would significantly alter the gaze patterns being observed throughout the experiment and would prevent accurate characterization of gaze behaviours during each stage of flight.

In line with previous work (5), we hypothesized that in visual flight rules (VFR) conditions, an increase in task difficulty will result in increased dwell time and fixation counts toward the external environment (i.e., front window), increased cognitive tunneling (i.e., frequency and duration), and an overall reduction in the dispersion and complexity of visual scan patterns as indicated by entropy measures. It was expected these changes in gaze behaviour reflect a greater need to allocate more time and attention toward fewer, more important task relevant AOIs during difficult conditions. It was also expected that the changes in gaze measures would be associated with reduced flight performance parameters and correlate with the pilot’s level of experience (i.e., flight hours) and SA (48; 51; 54).

## Methods

### Participants

Twenty-four participants (male: 14; age range: 19-40 years, mean= 22 years old, SD= 4 years) were recruited from the student and alumni populations at the University of Waterloo. All participants were current aviation students or other individuals who had obtained at least their private pilot’s license (PPL). All participants had normal or corrected-to-normal vision and had not been previously diagnosed with a neuropsychiatric/neurological disorder or learning disability. Participation in the study was voluntary, and participants received $25/hour as remuneration. The study’s protocol was approved by the University of Waterloo Research Ethics Board Committee (#43564), performed in accordance with the 2008 Declaration of Helsinki, and consent was obtained prior to beginning the protocol.

### Experimental Setup and Apparatus

**Figure 1.**
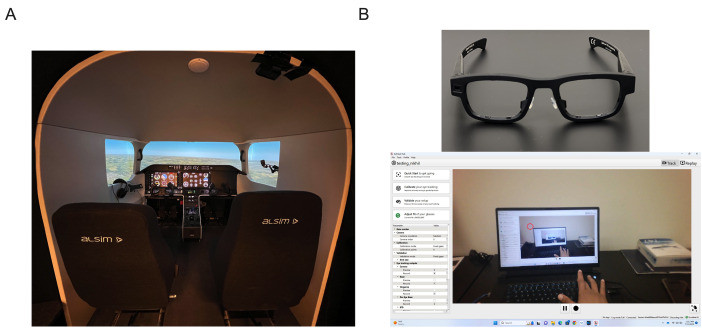
Illustration of the simulator and eye tracking equipment set-up. The ALSIM simulator was set up as a single-engine aircraft, controlled with the yolk, throttle lever, and rudders pedals (A). Participants also used a headset (hanging on the left window)to make ATC calls (A). The AdHawk glasses (B) were worn by participants throughout the experimental session and were connected to the collection laptop (B) via a USB-C cord. The collection laptop used AdHawk software to calibrate the eye tracker and start and stop eye tracking data collection.

*Flight Simulator*. An AL250 ALSIM flight simulator (ALSIM, France) configured as a generic single engine aircraft that is representative of a Cessna 172 was used with the necessary instrument panel (steam gauge configuration), an avionics/GPS system, an audio/lights panel, a breaker panel, and a Flight Control Unit (FCU) (see Figure 1). Participants sat in a height-adjustable seat (left pilot seat) with their aviation headset plugged in for ATC (air traffic control) callouts. The field of view covered by the simulator was 250° by 49° via panoramic VFR-VR-HD projectors. The participants controlled the aircraft with a yoke, throttle lever, and rudder pedals. Stimuli presentation, and behavioural data collection and acquisition were controlled from the Instructor Station and Engineering pack (ALSIM, France).

*Eye Tracker.* MindLink eye-tracking glasses (AdHawk Microsystems Inc., Waterloo, ON, Canada) were used to track the participants’ eye and gaze movements (Figure 1). MindLink is a non-camera-based eye tracker embedded in a frame of eyeglasses that uses an ultra-compact micro-electromechanical system (MEMS) to track the eye and gaze movements (63). The eye tracker was operating at 250 Hz, transmitting the gaze data and the video of its front-facing camera (82° field of view, 1080p, 30 Hz) via the AdHawk eye tracking software to a laptop (60 Hz refresh rate, 1920 x 1080 pixels, Microsoft 11) visible only to the experimenter (Figure 1).

**Figure 2.**
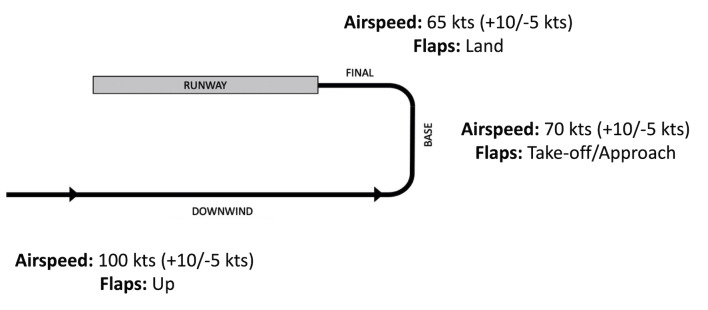
Illustration of the flight path and its respective phases of flight encountered in the experimental landing scenarios: Downwind, Base, Final Approach.

*Scenario and task*. Participants were tested in a single session (approx. 90 minutes). A visual screening was first completed including a visual acuity test using the Bailey-Lovie chart and a stereoacuity test using the Randot Stereo test (Stereo Optical Company, Inc.). Prior to commencing the experimental trials, a pilot briefing (completed by an instructor pilot) and practice trial was performed to familiarize the participants with the simulator environment and flight path (AL 250, ALSIM, France). The briefing also covered the segments of the flight that included the downwind (antiparallel), base (perpendicular) and final approach (in line with runway) phases of flight, which are primarily determined based on the spatial orientation of the aircraft relative to the designated runway and the required tasks associated with each leg of flight (24) (Figure 2).

**Figure 3.**
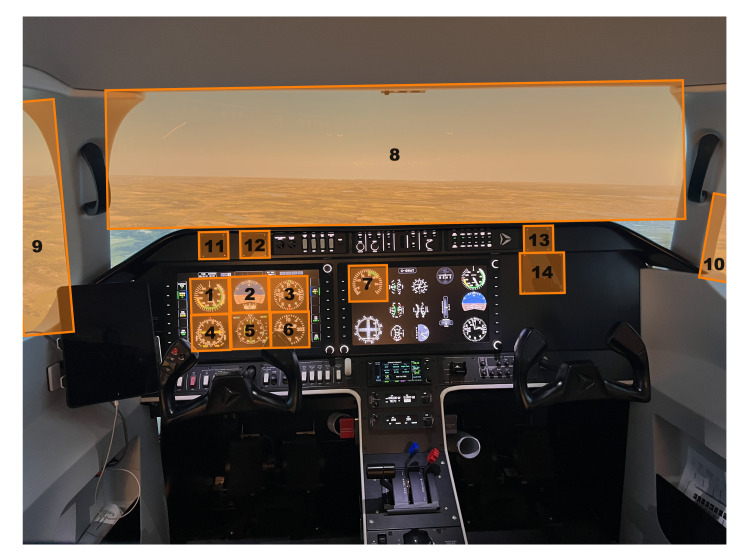
Illustration of the visual stimuli employed in the AL250 flight simulator environment. The participants point of view of the cockpit replicated that of a pilot flying a Cessna 172, pre-set for a downwind-to-base-to-final approach to Waterloo International Airport, Breslau, Ontario, CA. The orange boxes represent the ten main areas of interest used in the gaze analyses. These include the airspeed (1), attitude (2), altimeter (3), VOR (4), heading (5), vertical speed (6) and power (7) indicators, as well as the front (8), left (9), and right windows (10).

The experimental landing scenarios were programmed in the flight simulator environment, flying into the Region of Waterloo International Airport (CYKF; Runway 26), Breslau, Ontario, Canada. Participants were asked to complete a total of 8 customized landing trials while their eye and head movements were recorded. The landing challenges were pseudo-randomized into 4 easy (i.e., high visibility [>20 miles] and low wind [0 kts, 0ᵒ] conditions), and 4 difficult (i.e., high visibility [>20 miles] and high wind [26 kts, 230ᵒ] conditions) trials (i.e., 2 task difficulties/scenarios with 4 attempts/trials in each scenario). Note that the wind speed and direction for the difficult trial produced a cross wind component of 13 kts, while providing a tail wind of 22.5 kts during the downwind stage of flight and a head wind of 22.5 kts during the final approach stage of flight. This inherently allowed the downwind leg of flight to be completed faster, while the final approach stage of flight was likely to be completed much slower, compared to the easy condition. All participants received identical environmental configurations. Figure 3 illustrates the visual conditions of the simulated scenario. Each trial was pre-set to start as a downwind-to-base-to-final approach to the airport at an altitude of 2017 ft at sea level (airport altitude 1054 ft), 1 nautical mile away from the downwind runway threshold with flaps and trim set to zero, and at a starting speed and power of approximately 110 kts and 2000 rpm, respectively. The simulated landing task involved VFR conditions where visibility was high, which represents one of the most basic landing scenarios that novice pilots are faced with during training. This allowed for the extension of previous work that used similar paradigms and more advanced aircraft configurations (i.e., helicopter simulators, A320 flight simulators, larger aircrafts with glass cockpit displays) (5; 12; 19; 54; 55). This was particularly important as the present work recruited low-time pilots (i.e., <300 hours of flight time).

At the start of each trial, the participant manually initiated the landing scenario once 9-point eye-tracking calibration and validation procedures were completed by the examiner (average gaze error <2°). The goal of the task was to land the plane as smoothly and accurately as possible relative to the center of the 500 ft markers near the start of the runway. The trial was terminated after the participant brought the plane to a complete stop, or if the landing was deemed unsuccessful (i.e., plane crash or plane landed off the runway). The participant was then asked to complete the Situational Awareness Rating Technique (SART) questionnaire to gauge their subjective opinion on various domains related to task difficulty, as well as the supply and demand of attentional resources required during task performance (53).

### Data Reduction

Gaze data were post-processed offline using a custom-made script that used the 3D gaze vectors provided by the AdHawk software for saccade and fixation detection. The saccade detection algorithm was based on the algorithm proposed originally by Nyström and Holmqvist (39) with some modifications to work on the current data captured at 250 Hz. Unlike the original method that uses a fixed saccade-peak-velocity threshold for detecting the saccade candidates, we used a low-pass filtered version of the velocity signal (rolling average with a 50-sample window) to increase the threshold in the noisy regions of the data. After classifying the eye movements into saccades and fixations, the average of the gaze sample during each fixation was taken as the fixation position. Eye-movement traces were visualized by the experimenter and played back at a slowed speed superimposed over the video displaying the simulator environment. The task environment was discretized using a custom code by organizing the simulator environment into ten areas of interest (AOIs) (Figure 3). The AOIs were manually defined to represent seven main gauges of interest within the cockpit including, airspeed (1), attitude (2), altimeter (3), VHF Omni Range (VOR) (4), heading (5), vertical speed (6) and power (7). Three additional AOIs were also defined outside the cockpit including, the front window (8), the left window (9), and the right window (10). Fixations found outside these AOIs were defined as a non-area of interest and excluded from the analysis (<4%). The current study focused on primary saccades, thus microsaccades (<1°) were excluded from analysis (34). Trials with missing data (i.e., loss of signal >30%) (~4% of trials) and outliers for each of the dependent variables (i.e., >1.5 the interquartile range around the first and third quartiles) (~2% of trials) were removed.

### Performance, Gaze, and Situation Awareness Analysis

We evaluated the flight performance, gaze behaviour, and subjective level of situation awareness across the two task difficulties (easy, difficult). Flight was assessed across two domains: 1) Landing Performance and 2) Aircraft Control. Landing Performance included completion time (sec; duration of time from the start of the landing scenario to the plane coming to a complete stop on the runway), landing accuracy (degrees; the difference between the center of the plane and the center of the 500 ft runway marker at point of touchdown), and landing hardness (feet per minute, or fpm; the rate of decent at point of touchdown). Aircraft Control included the mean, standard deviation (i.e., variability) and root mean square error (RMSE) of the aircraft airspeed (i.e., the average difference between the reference optimal airspeed [downwind= 100 kts, base= 70 kts, final= 65 kts] and the participants’ observed airspeed, kts) and vertical speed (the average difference between the reference optimal vertical speed [final= -325 kts] and the participants’ observed vertical speed, fpm).

In line with previous work, gaze behaviour was examined using traditional gaze measures, as well as static and dynamic entropy-based analyses (6; 5; 64). Traditional gaze-based analysis was completed using the ten AOIs (Figure 3) that were discretized during pre-processing using a custom script. Specifically, we examined dwell time, average dwell duration, and dwell rate across all AOIs. Dwell time was defined as the total duration spent within a given AOI as function of total flight time, reported here as a percentage (i.e., with respect to total time). Average dwell duration was defined as the average duration of time (msec) of all uninterrupted dwells within a given AOI. Dwell rate was defined as the number of fixations that occurred within a given AOI over a given period of time (i.e., dwells/sec). Lastly, blink rate was defined as the number of blinks that occurred over a given period of time (i.e., count/sec).

The static entropy-based analysis was completed using the ten AOIs (Figure 3) that were discretized during pre-processing. Eye fixations in the ten AOIs were assigned a number from 1 to 10 indicating the AOI where the eyes fixated. A sequence of fixation locations was then generated for each trial. Custom scripts were written in Python to compute both SGE and GTE (Ayala et al., 2021; 5), which were then normalized (**Equation 1**) (45).

**Equation 1:**
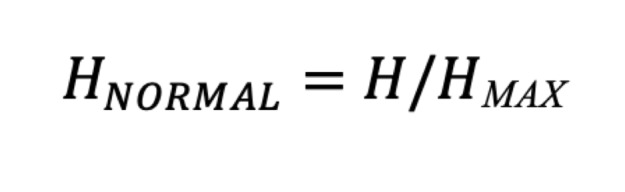


SGE was computed by first producing a vector, **V**, of length 10, where **V_i_** was the total number of fixations at AOI **i**. **V** was then divided by the total number of fixations in the sequence, so that **V_i_** was the probability of a fixation landing at AOI **i**. The probability vector **V** was then applied to **Equation 2** (45).

**Equation 2:**
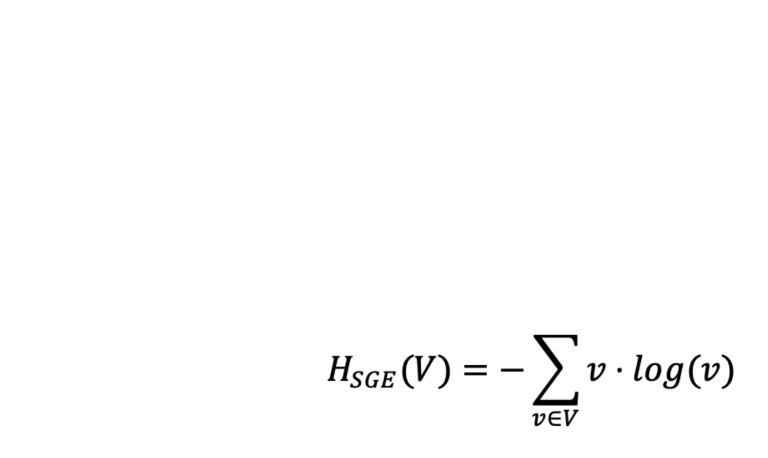


GTE was computed by first creating a 10x10 transition matrix, **M**, where **M_i,j_** was the total number of transitions from AOI **i** to AOI **j**. Each row, **M_i,∗_**, was divided by the sum of row **i**, so that **M_i,∗_** represented the probability of fixation transition from AOI **i** to any of the ten AOIs. Finally, GTE was computed using **Equation 3** (14), applying the transition matrix **M** and the probability vector **V.**

**Equation 3:**
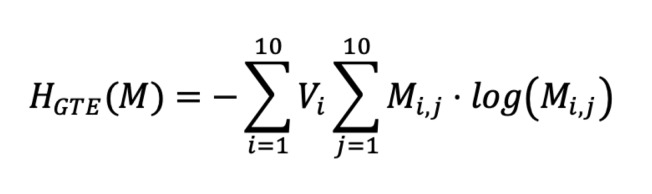


The dynamic entropy-based analysis developed by Ayala et al. (5) was completed using a 10 second average sliding window to show the probability of a fixation being inside the cockpit over the length of the trial, P(inside). The choice of a 10 second sliding window was based on previous work that initially arbitrarily chose a 30 second sliding window but found it did not have sufficient resolution in detecting the observed characteristic cycling of gaze behaviour (5). Notably, this ‘gaze tunneling bout analysis’ is specifically different from “tunnel vision” in that we are not assessing if there is a loss/reduction of useful peripheral vision. Instead, gaze tunneling is particularly interested in quantifying the reduction in gaze transitions from the external environment to the cockpit gauges; a behaviour which has been tied to reduced pilot performance as a result of poor instrument scanning behaviour (1; 2; 59). Nevertheless, to reduce the potential for confusion between tunnel vision and gaze tunneling, we have chosen to refer to this dynamic entropy-based analysis from here on out as cognitive tunneling bout analysis. Cognitive tunneling is a phenomenon in which an individual jeopardizes their ability to perceive all pertinent information in their environment because of a tendency to focus on a singular AOI (9; 22; 56). As such, it more accurately reflects the gaze behaviour that the dynamic-entropy based analysis captures.

To examine this phenomenon dynamically Ayala and colleagues assigned a binary number based on whether the fixation was inside or outside the cockpit. P(inside) was then computed as the number of fixations inside the cockpit divided by the total number of fixations in the 10 second window. When P(inside) was equal to 1, the participant was continuously fixating inside the cockpit for at least 10 seconds. When P(inside) was equal to 0, the participant was continuously fixating on the outside scenery for at least 10 seconds (i.e., cognitive tunneling). This was employed to objectively monitor the temporal dynamics of gaze behaviour, with a focus on how attention was deployed inside and outside the cockpit. Cognitive tunneling is an important behaviour to quantify in pilot scanning. In this context, it refers to the absence of pilot scanning toward other AOIs that have pertinent information regarding aircraft control. As such, it may impact pilot performance as well as situation awareness (1; 2; 5; 9; 59). A cognitive tunneling ‘bout’ was defined as a period of time in which fixations remained entirely outside of the cockpit for at least 10 seconds. These bouts were detected as connected components (subsequent values) of zeros in the probability time series. Number of bouts was defined as the number of instances a cognitive tunneling bout was detected within a trial. Bout duration (sec) was defined as the average duration of all bouts that occurred in a trial. Total bout time (sec) was defined as the sum of all bout durations to quantify the total time individuals demonstrated gaze behaviours reflective of cognitive tunneling.

Situation awareness was assessed using a subjective questionnaire and a scenario probe. The SART questionnaire (53) is a post-trial self-report questionnaire that uses a 7-point Likert scale (1=Low; 7=High) across 10 dimensions of situation awareness. Note that this is collapsed into three larger dimensions of attentional demands, attentional supply, and situation understanding. These ratings are then combined to calculate a measure of situation awareness (SA).

**Equation 4:**
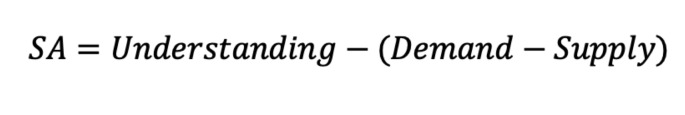


### Statistical Analysis

Aircraft control (i.e., airspeed), traditional gaze measures, and static entropy measures for successful trials were analyzed using a two-way repeated measures ANOVA with Phase of Flight (Downwind, Base, Final) and Task Difficulty (Easy, Difficult) as the independent variables. This specific analysis provides in-depth insight into the effect of increasing task difficulty across various stages of flight. Additionally, landing performance, aircraft control (i.e., vertical speed), dynamic entropy measures and subjective situation awareness scores for successful trials were analyzed using a one-way repeated measures ANOVA with Task Difficulty (Easy, Difficult) as the only independent variable. Note that the vertical speed parameter of aircraft control was included in this ANOVA model as this was only measured for the final approach phase of flight. All ANOVAs were performed with an alpha level set at 0.05. The Bonferroni post hoc correction for multiple comparisons was applied for all post hoc analyses following the repeated measure ANOVAs to determine significant differences between variables. A secondary analysis was conducted using a linear mixed model to assess the effect of flight hours (between-subject expertise measure) on gaze behaviour, situation awareness, and performance measures, with Task Difficulty as the repeated measures variable. The linear mixed model analysis was also completed to examine the effect of gaze behaviour on situation awareness and performance measures.

## Results

Participant demographics with respect to flight hours showed that on average participants had 180 flight hours (SD= 75) with a minimum of 51 hours and a maximum of 280 hours.

### The effects of task difficulty on landing performance and aircraft control

**Figure 4.**
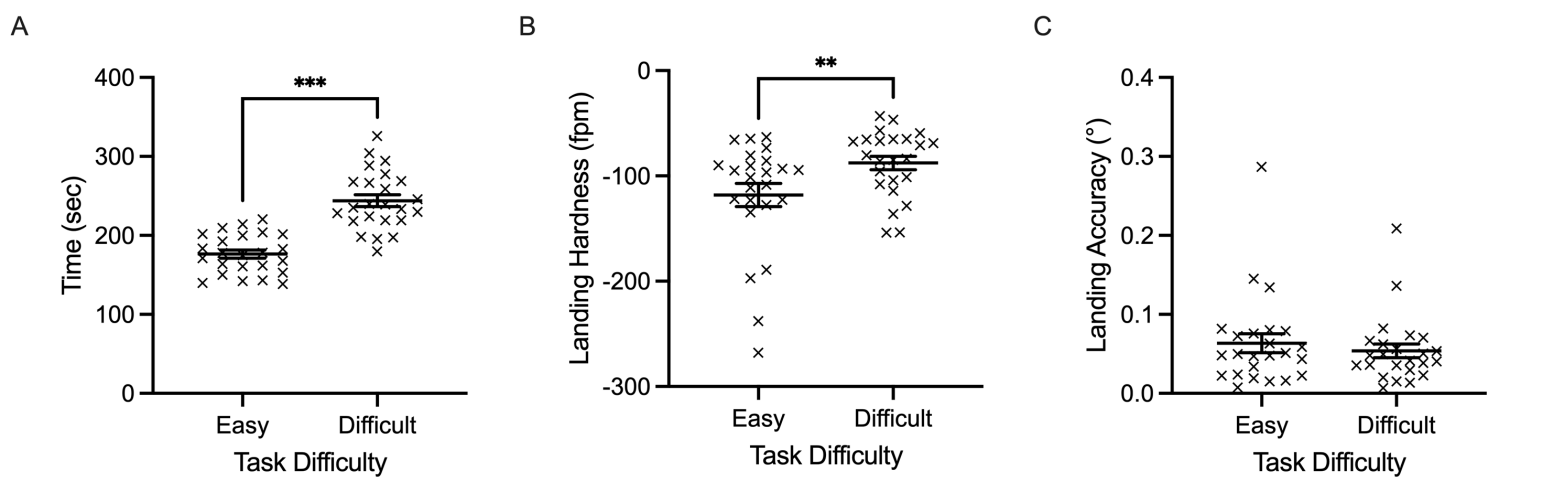
Individual data points and their respective group means for completion time (A), landing hardness (B), and landing accuracy (C) are demonstrated for easy and difficult conditions. Error bars represent SEM. *p ≤0.05, **p ≤0.01, ***p ≤0.001.

**Landing performance.** Completion time (sec) produced a main effect of task difficulty, F(1, 23)= 294.054, p<0.001, 𝜂_p_^2^= 0.927. Easy trials were completed significantly quicker (X= 176.36 sec, SD= 25.02) than difficult trials (X= 243.98 sec, SD= 37.04) (Figure 4A). Landing hardness produced a main effect of task difficulty, F(1, 23)= 11.594, p=0.003, 𝜂_p_^2^= 0.345. Specifically, easy trials were associated with significantly increased landing hardness (fpm) (X= -118.12 fpm, SD= 53.48) compared to difficult trials (X= -87.72 fpm, SD= 31.53) (Figure 4B). Lastly, landing accuracy did not significantly change between task difficulty, F(1, 23)= 1.104, p=0.304 (Figure 4C).

**Figure 5.**
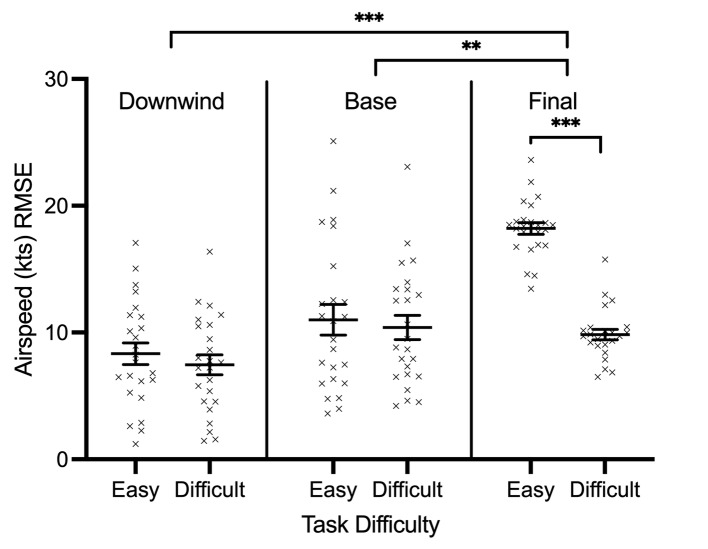
Individual data points and their respective group means for average airspeed RMSE are demonstrated for easy and difficult conditions across all downwind, base, and final approach stages of flight. Error bars represent SEM. *p ≤0.05, **p ≤0.01, ***p ≤0.001.

**Aircraft control**. Mean airspeed (kts) demonstrated a main effect of phase of flight, F(2, 46)= 519.682, p<0.001, 𝜂_p_^2^= 0.958, task difficulty, F(2, 46)= 33.877, p<0.001, 𝜂_p_^2^= 0.596, and an interaction involving phase of flight and task difficulty, F(2, 46)= 46.665, p<0.001, 𝜂_p_^2^= 0.670. Mean airspeed decreased significantly across each stage of flight (downwind: X=93.52 kts, SD= 3.58; base: X=77.55 kts, SD= 5.93; final: X=62.69 kts, SD=2.50). Moreover, difficult trials were associated with an increase in mean airspeed (X= 79.41 kts, SD= 3.82) compared to easy trials (X= 76.43 kts, SD= 3.17). However, post-hoc comparison of the interaction indicates that the task difficulty effect was only significant during the final approach phase of flight (easy: X= 58.71 kts, SD= 2.66; difficult: X= 66.66 kts, SD= 3.28). Airspeed variability (kts) also yielded a main effect of flight phase, F(2, 46)= 16.880, p<0.001, 𝜂_p_^2^= 0.423, task difficulty, F(2, 46)= 99.273, p<0.001, 𝜂_p_^2^= 0.812, and an interaction involving flight phase and task difficulty, F(2, 46)= 10.917, p<0.001, 𝜂_p_^2^= 0.322. Specifically, airspeed variability increased significantly across each stage of flight (downwind: X= 3.39 kts, SD= 1.62; base: X= 5.98 kts, SD= 2.79; final: X= 12.94 kts, SD= 1.67). Easy trials had significantly higher airspeed variability compared to difficult trials (easy: X= 8.84 kts, SD= 0.98; difficult: X= 6.04 kts, SD= 0.65). However, the phase of flight by task difficulty interaction indicated that this was specific to the final stage of flight (easy: X= 16.80 kts, SD= 2.12; difficult: X= 9.07 kts, SD= 1.48). Lastly, airspeed RMSE demonstrated a main effect of phase of flight, F(2, 46)= 13.123, p=0.001, 𝜂_p_^2^= 0.363, task difficulty, F(2, 46)= 107.577, p<0.001, 𝜂_p_^2^= 0.824, and an interaction involving phase of flight and task difficulty, F(2, 46)= 42.813, p<0.001, 𝜂_p_^2^= 0.651. Figure 5 illustrates a significant increase in airspeed RMSE across each phase of flight (downwind: X= 7.89 kts, SD= 3.49; base: X= 10.69 kts, SD= 5.09; final: X= 14.03 kts, SD= 1.90). Though the main effect of task difficulty suggests easy trials had increased airspeed RMSE (X= 12.52 kts, SD= 1.88) compared to difficult trials (X= 9.23 kts, SD= 1.61), the interaction shows that this is only the case during the final approach stage of flight (easy: X= 18.22 kts, SD= 2.25; difficult: X= 9.84 kts, SD= 2.02) (Figure 5).

**Figure 6.**
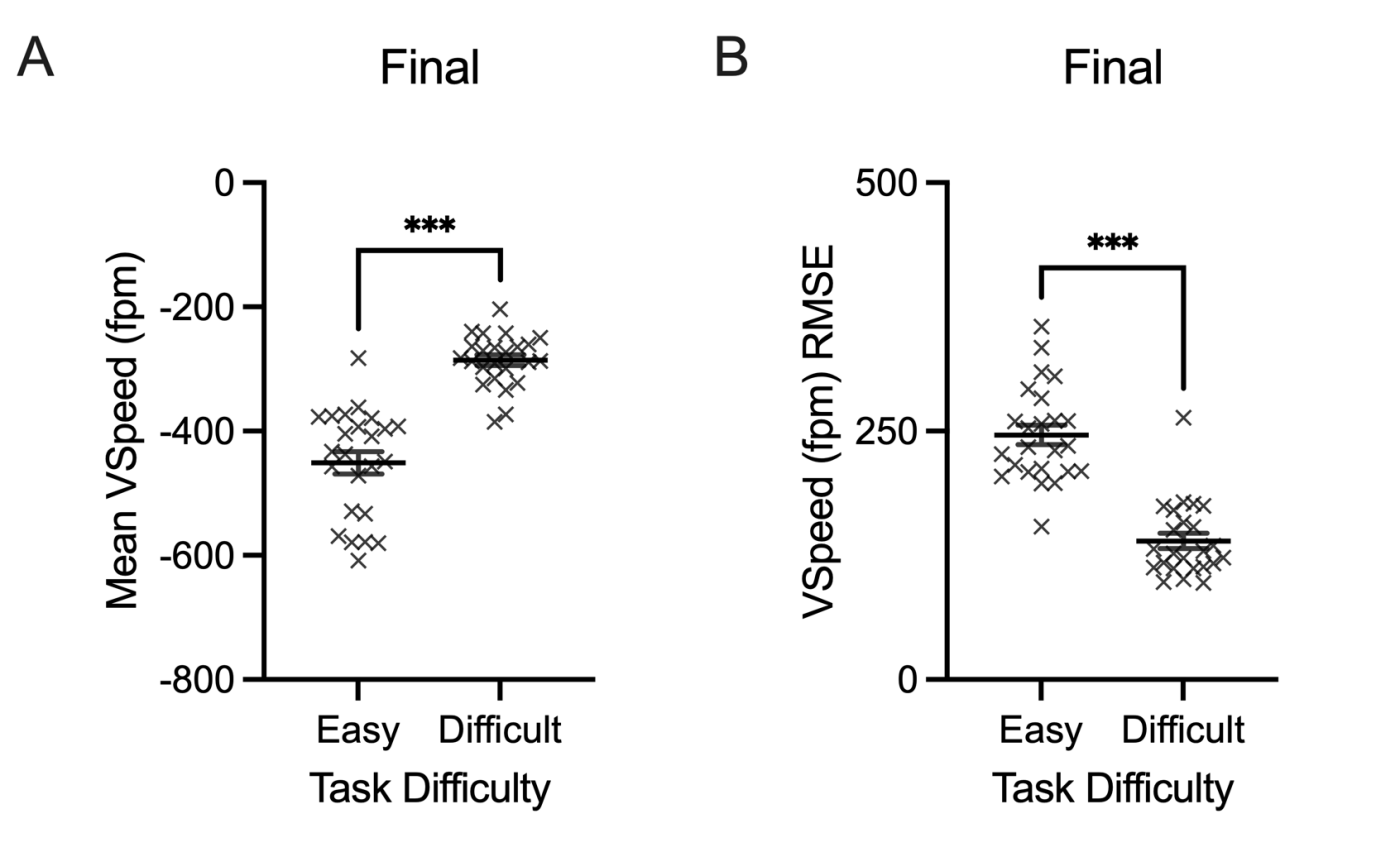
Individual data points and their respective group means for vertical speed (VSpeed) (kts) (A), and VSpeed root mean square error (RMSE) (B) are demonstrated for easy and difficult conditions across the final approach stage of flight. Error bars represent SEM. *p ≤0.05, **p ≤0.01, ***p ≤0.001.

Mean vertical speed (fpm) yielded a main effect of task difficulty during the final approach phase of flight, F(1, 23)= 146.312, p<0.001, 𝜂_p_^2^= 0.864. Figure 6A demonstrates how difficult trials are associated with significantly lower mean vertical speed (X= -285.85 fpm, SD= 41.85) compared to easy trials (X= -451.00 fpm, SD= 87.12). Vertical speed variability (fpm) also demonstrated a main effect of task difficulty during the final approach phase of flight, F(1, 23)= 124.816, p<0.001, 𝜂_p_^2^= 0.844. Specifically, vertical speed variability was significantly higher during easy trials (X= 246.15 fpm, SD= 48.14) compared to difficult trials (X= 139.52 fpm, SD= 37.36). Vertical Speed RMSE demonstrated a main effect of task difficulty, F(1, 23)= 65.785, p<0.001, 𝜂_p_^2^= 0.741. Figure 6B illustrates how difficult trials are associated with a reduced RMSE (X= 154.43 fpm, SD= 7.18) compared to easy trials (X= 286.53 fpm, SD= 15.88).

### The effects of task difficulty on gaze behaviour

**Figure 7.**
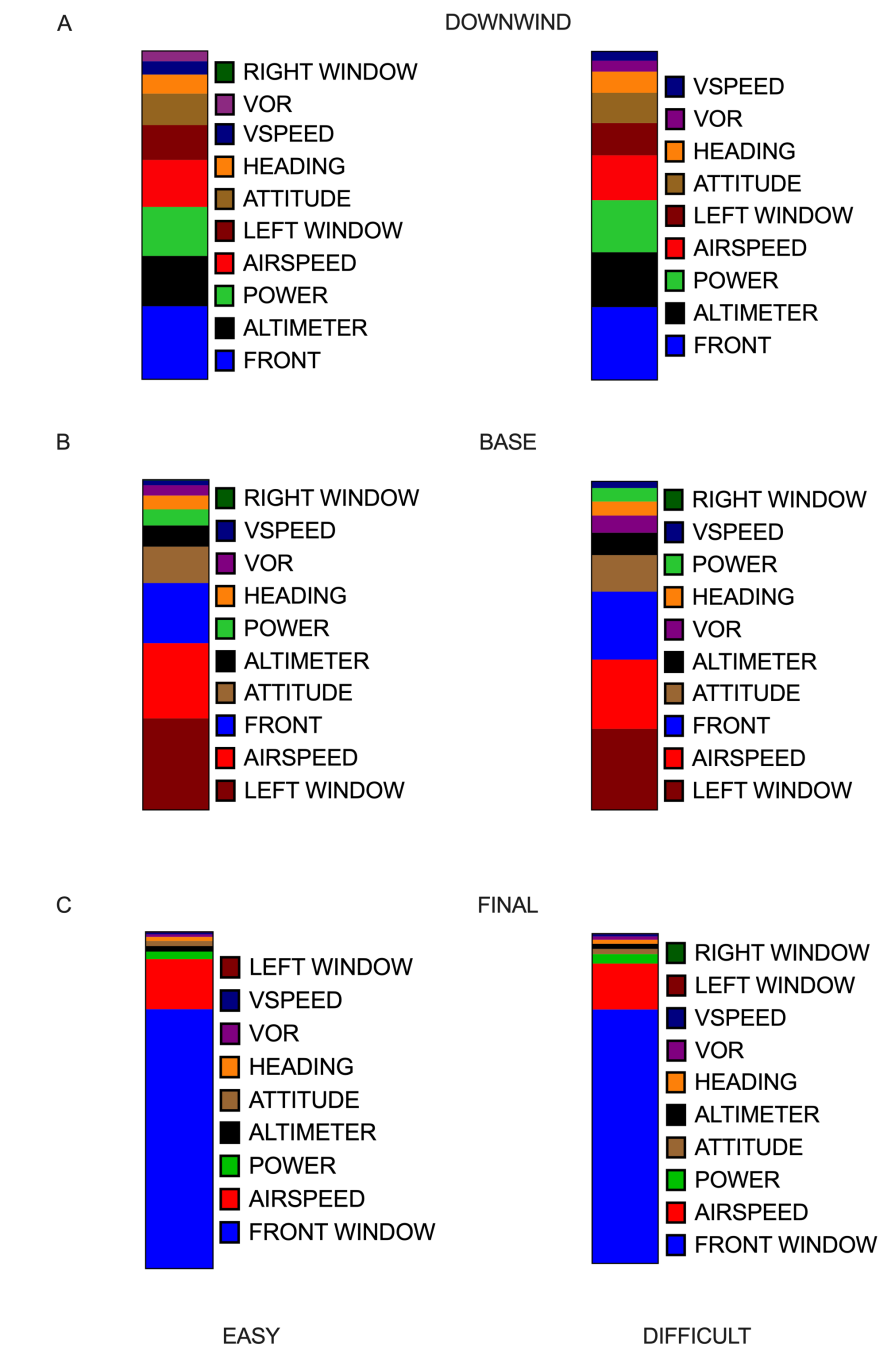
Candy-bar plots illustrate the overall dwell time (%) allotments across all AOIs during their respective phases of flight (A, B, C) for easy (left panels) and difficult (right panels) conditions.

*Traditional Gaze Metrics.* Dwell time (%) means and standard deviations for all AOIs across all task conditions are reported in Table 1. Dwell time (%) revealed significant changes across several AOIs associated with flight phase and task difficulty (Figure 7). Dwell time on the airspeed AOI demonstrated a main effect of flight phase, *F*(2,46)= 12.881, *p*<0.001, 𝜂_p_^2^=0.359, and task difficulty, F(1, 23)= 4.693, *p*=0.041, 𝜂_p_^2^=0.169. Specifically, airspeed AOI dwell time decreased on average in the difficult trials (-0.78%) relative to the easy trials. Airspeed AOI dwell time was at its highest during the base phase of flight, followed by the final, and downwind phases of flight. Attitude AOI demonstrated a main effect of phase, *F*(2,46)= 32.560, *p*<0.001, 𝜂_p_^2^=0.586. Specifically, dwell time for the attitude gauge was at its highest during the base leg of flight, followed by the downwind, and final phases of flight. There was a main effect of flight phase for the Altimeter AOI, *F*(2, 46)= 46.605, p<0.0001, 𝜂_p_^2^=0.670 and, *ps*<0.001, and for the power AOI, *F*(2, 46)= 40.179, p<0.0001, 𝜂_p_^2^=0.636. Dwell times on these AOIs decreased significantly from the downwind to the base and then final phases of flight. VOR AOI showed a main effect of flight phase, *F*(2, 46)= 7.448, *p*=0.007, 𝜂_p_^2^=0.245, as well as an interaction between phase and task difficulty, *F*(2, 46)=5.829, *p*=0.006, 𝜂_p_^2^=0.202. Decomposition of the interaction revealed that the difficult condition was associated with a longer dwell time (+1.79%) on the VOR AOI during the base phase of flight, *t*(23)=-2.612, *p*=0.016. Dwell times on the heading and vertical speed AOIs revealed a main effect of phase *F*(2, 46)=6.093 and 9.298, *p*=0.010 and 0.003, 𝜂_p_^2^=0.209 and 0.288, respectively. Dwell times on both AOIs decreased from the initial downwind phase to the base phase, and again in the final phase of flight. Front window AOI dwell time demonstrated a main effect of phase, *F*(2, 46)=604.499, *p*<0.001, 𝜂_p_^2^=0.963. Dwell time through the front window increased significantly during the final phase of flight compared to both the downwind and base phases of flight. Left window AOI dwell time demonstrated a main effect of phase, *F*(2, 46)=109.765, *p*<0.001, 𝜂_p_^2^=0.827, as well as a main effect of task difficulty, *F*(1, 23)=6.211, *p*=0.020, 𝜂_p_^2^=0.213. Specifically, dwell time on left window AOI was longest during the base phase of flight, which was followed by the downwind phase and the final approach phase of flight. Left window AOI dwell time also decreased significantly in the difficult condition (-0.94%) compared to the easy condition. Right window AOI dwell time did not show any significant changes due to flight phase or task difficulty. The significant changes in the distribution of attention (i.e., dwell time %) observed between easy and difficult conditions across all phases of flight are illustrated in figure 7.

**Table:**
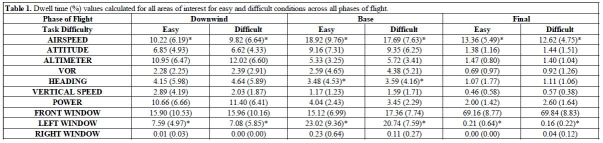


Note. Mean (standard deviation) dwell time (%) values across all areas of interest and task difficulty levels (i.e., easy, difficult). Significant changes between easy and difficult conditions are reported via *p ≤0.05, **p ≤0.01, ***p ≤0.001.

Dwell rate means and standard deviations for all AOIs across all task conditions are reported in Table 2. Dwell rate (dwells/sec) was shown to be modulated by phase of flight and task difficulty to varying extents across several AOIs. Airspeed dwell rate showed a main effect of phase, *F*(2, 46)=5.891, *p*=0.005, 𝜂_p_^2^=0.204. Dwell rate at the airspeed gauge was highest during the downwind phase of flight and decreased significantly in the base and final phases of flight. Attitude dwell rate also demonstrated a main effect of phase, *F*(2, 46)=13.400, *p*<0.001, 𝜂_p_^2^=0.368. Attitude dwell rate was highest during the final phase of flight, which was followed by the downwind phase and then the base phase of flight. Altimeter dwell rate demonstrated a main effect of phase, *F*(2, 46)=14.324, *p*<0.001, 𝜂_p_^2^=0.384. Dwell rate toward the altimeter gauge was highest during the final phase of flight, which was significantly different from the base and downwind phases of flight. Front window dwell rate demonstrated a main effect of phase, *F*(2, 46)=97.324, *p*<0.001, 𝜂_p_^2^=0.809, which was associated with a significant reduction in dwell rate from the downwind phase of flight to the base phase, then again from the base phase to the final phase of flight. Notably, there was a phase by task difficulty interaction, *F*(2, 46)=4.896, *p*=0.020, 𝜂_p_^2^=0.176. Decomposition of the interaction did not reveal any significant differences across the conditions, but it is worth noting that front window dwell rate was slightly higher (+0.01 dwells/sec) in the difficult condition than the easy condition during the downwind phase. In contrast, all other phases of flight demonstrated lower front window dwell rates for the difficult condition compared to the easy condition. Left window dwell rate yielded a main effect of phase, *F*(2, 46)=10.405, *p*=0.002, 𝜂_p_^2^=0.311. The downwind phase of flight had the highest left window dwell rate, which was significantly lower in the base and final phases of flight. Right window dwell rate was the only AOI to demonstrate a main effect of task difficulty, *F*(1, 23)=4.844, *p*=0.038, 𝜂_p_^2^=0.174. Specifically, the difficult condition was associated with an increase in dwell rate toward the right window (+0.04 dwells/sec) compared to the easy condition. All other AOIs did not demonstrate any significant changes due to phase of flight or task difficulty (*ps*>0.083).

**Table:**
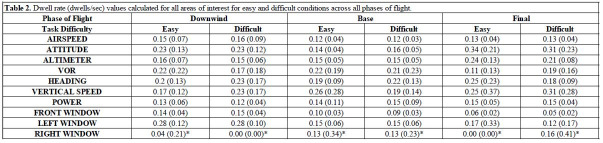


Note. Mean (standard deviation) dwell rate (dwells/sec) values across all areas of interest and task difficulty levels (i.e., easy, difficult). Significant changes between easy and difficult conditions are reported via *p ≤0.05, **p ≤0.01, ***p ≤0.001.

Dwell duration means and standard deviations for all AOIs across all task conditions are reported in Table 3. Average dwell duration demonstrated main effect of flight phase that was specifically shown for a limited number of AOIs including, Attitude, *F*(2, 46)=22.714, *p*<0.001, 𝜂_p_^2^=0.497, Altimeter, *F*(2, 46)=10.820, *p*<0.001, 𝜂_p_^2^=0.320, power, *F*(2, 46)=8.598, *p*=0.001, 𝜂_p_^2^=0.290, and the Front window, *F*(2, 46)=58.488, *p*<0.001, 𝜂_p_^2^=0.718. Attitude average dwell durations were longest during the base phase of flight, which was significantly longer than both the downwind and final approach phases of flight. Altimeter dwell durations were longest during the downwind phase of flight, then significantly decreased during the final phase of flight. Power dwell durations were longest during the downwind phase of flight and significantly decreased across each subsequent stages of flight (i.e., base and final approach). Front window dwell durations were longest during the final approach stage of flight, which became significantly shorter during each preceding phase of flight (i.e., base and downwind). All other AOIs did not demonstrate any significant changes as a result of phase of flight or task difficulty (*ps*>0.05).

**Table:**
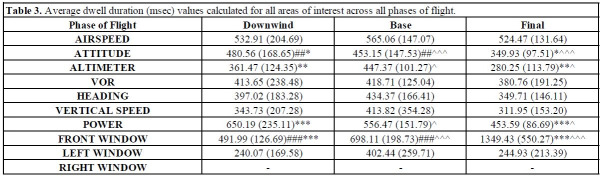


Note. Mean (standard deviation) average dwell duration (msec) values across all areas of interest and phases of flight (i.e., downwind, base, final). Significant changes between downwind and base phase of flight are reported via ^#^ *p* ≤0.05, ^##^ *p* ≤0.01, ^###^ *p* ≤0.001. Significant changes between base and final phase of flight are reported via ^^^ *p* ≤0.05, ^^^^ *p* ≤0.01, ^^^^^ *p* ≤0.001. Significant changes between downwind and final phase of flight are reported via ^*^ *p* ≤0.05,^**^ *p* ≤0.01, ^***^ *p* ≤0.001.

**Figure 8.**
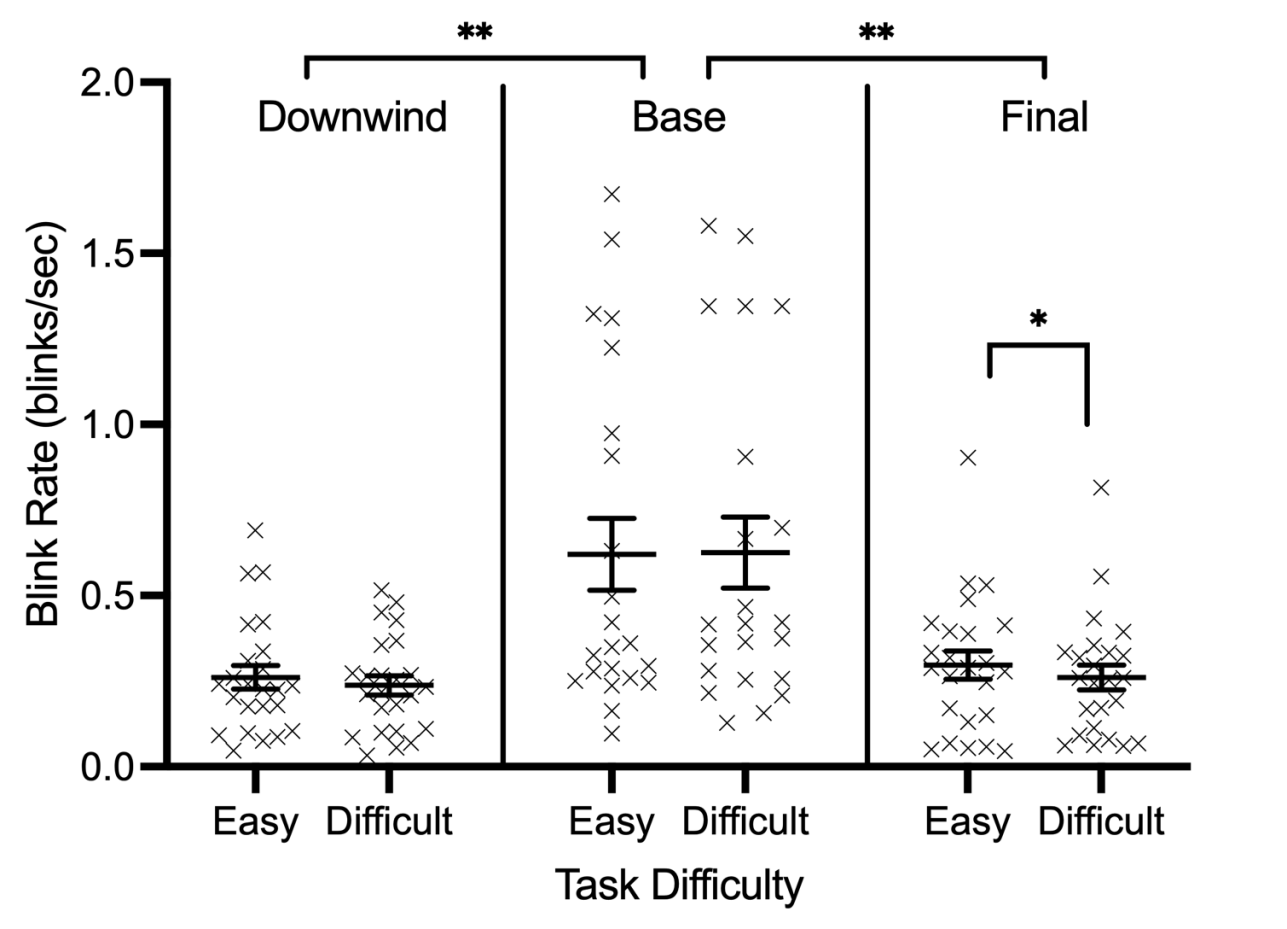
Individual data points and their respective group means for blink rate (blinks/sec) are demonstrated for each phase of flight. Error bars represent SEM. *p ≤0.05, **p ≤0.01, ***p ≤0.001.

Changes in blink rate (blinks/sec) across flight phases are shown in figure 8. Results demonstrated a main effect of flight phase *F*(2, 46)=11.609, *p*=0.001, 𝜂_p_^2^=0.335, and task difficulty, *F*(1, 23)=4.955, *p*=0.036, 𝜂_p_^2^=0.177, as well as an interaction between phase and task difficulty *F*(2, 46)=3.215, *p*=0.049, 𝜂_p_^2^=0.123 (Figure 8). The base phase of flight was associated with the highest blink rate (0.62 blinks/sec, SD= 0.47) compared to the downwind (0.25 blinks/sec, SD= 0.15) and final approach phases of flight (0.28 blinks/sec, SD= 0.19). Additionally, blink rate was significantly lower during the difficult condition (0.37 blinks/sec, SD= 0.17), compared to the easy condition (0.39 blinks/sec, SD= 0.17). Decomposition of the interaction revealed that blink rate was significantly lower during the difficult condition, specifically during the final phase of flight (easy: 0.29 blinks/sec, SD= 0.07; difficult: 0.26 blinks/sec, SD= 0.07), *t*(23)=2.245, *p*=0.035.

**Figure 9.**
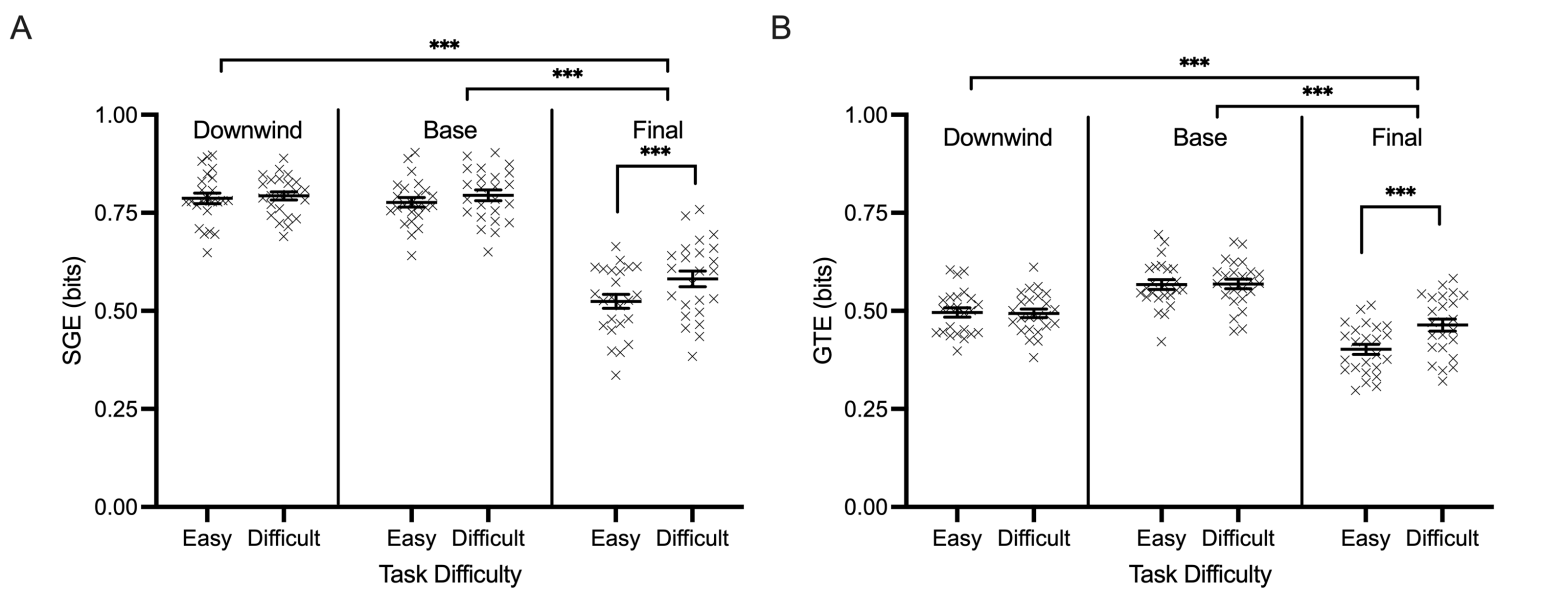
Normalized individual data points and their respective group means for stationary gaze entropy (SGE) (bits) (A) and gaze transition entropy (GTE) (B) are demonstrated for easy and difficult conditions each phase of flight (i.e., downwind, base, final approach) . Error bars represent SEM. *p ≤0.05, **p ≤0.01, ***p ≤0.001.

SGE and GTE means and standard deviations for all AOIs across all task conditions are reported in Table 4. SGE demonstrated a main effect of phase, *F*(2, 46)=137.971, *p*<0.001, 𝜂_p_^2^=0.857, task difficulty, *F*(1, 23)=14.628, *p*=0.001, 𝜂_p_^2^=0.389, and an interaction involving phase and task difficulty, *F*(2, 46)=6.054, *p*=0.005, 𝜂_p_^2^=0.208. SGE was highest during the initial downwind phase of flight and decreased significantly during the final approach stage. In general, the more difficult task condition was associated with greater fixation dispersion. However, decomposition of the phase by task difficulty interaction specifically revealed that SGE differed significantly between easy and difficult conditions (~0.19 bits) during the final approach phase of flight, *t*(23)=-4.292, *p*<0.001 (Figure 9A). GTE demonstrated similar main effects of flight phase, *F*(2, 46)=33.413, *p*<0.001, 𝜂_p_^2^=0.592, task difficulty, *F*(1, 23)=19.401, *p*<0.001, 𝜂_p_^2^=0.458, and an interaction involving phase and task difficulty, *F*(2, 46)=15.943, *p*<0.001, 𝜂_p_^2^=0.409. GTE was highest during the base phase of flight, which was significantly lower during the downwind phase of flight and the final approach stage of flight. The difficult condition was associated with a higher GTE. However, decomposition of the interaction involving phase and task difficulty showed that GTE was significantly different between the easy and difficult task conditions (~0.21 bits) during the final phase of flight only, *t*(23)=-5.816, *p*<0.001) (Figure 9B).

**Table:**
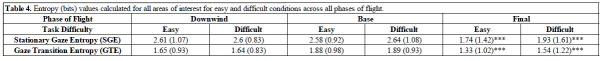


Note. Mean (standard deviation) entropy (bits) values across all phases of flight (i.e., downwind, base, final) and task difficulty levels (i.e., easy, difficult). Significant changes between easy and difficult conditions are reported via *p ≤0.05, **p ≤0.01, ***p ≤0.001.

**Figure 10:**
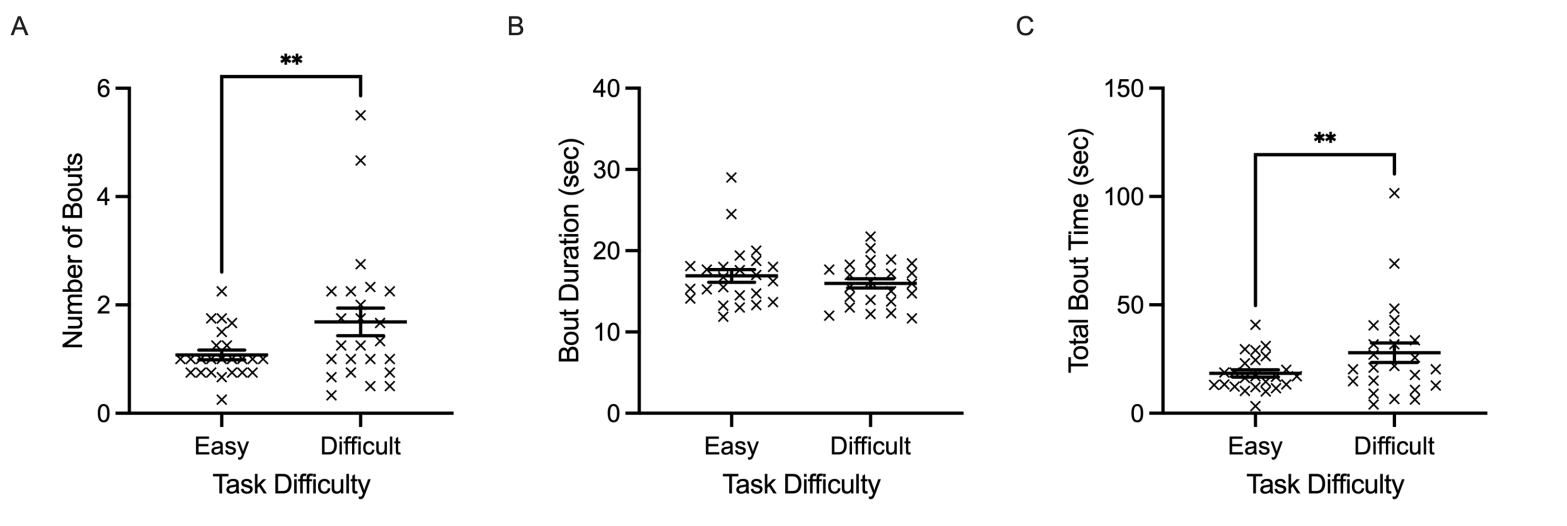
Individual data points and their respective group means for number of cognitive tunneling bouts (A) average bout duration (B), and total cognitive tunneling bout time (C) are demonstrated for easy and difficult conditions. Error bars represent SEM. *p ≤0.05, **p ≤0.01, ***p ≤0.001.

*Dynamic gaze behaviour.* Number of cognitive tunneling bouts revealed a main effect of task difficulty, *F*(1, 23)=10.360, *p*=0.004, 𝜂_p_^2^=0.311. Specifically, number of bouts was significantly higher in the difficult condition (X=1.69, SD= 1.24) compared to the easy condition (X=1.07, SD= 0.43) (Figure 10A). Total bout time (sec) also demonstrated a main effect of task difficulty, *F*(1, 23)=8.006, *p*=0.010, 𝜂_p_^2^=0.258. The difficult condition was associated with significantly longer total bout time (X=27.91, SD= 21.96), compared to the easy condition (X=18.41, SD= 8.39) (Figure 10C). Average bout duration was not significantly modulated by task difficulty (*p*=0.275) (Figure 10B).

### The effects of task difficulty on situation awareness

**Figure 11.**
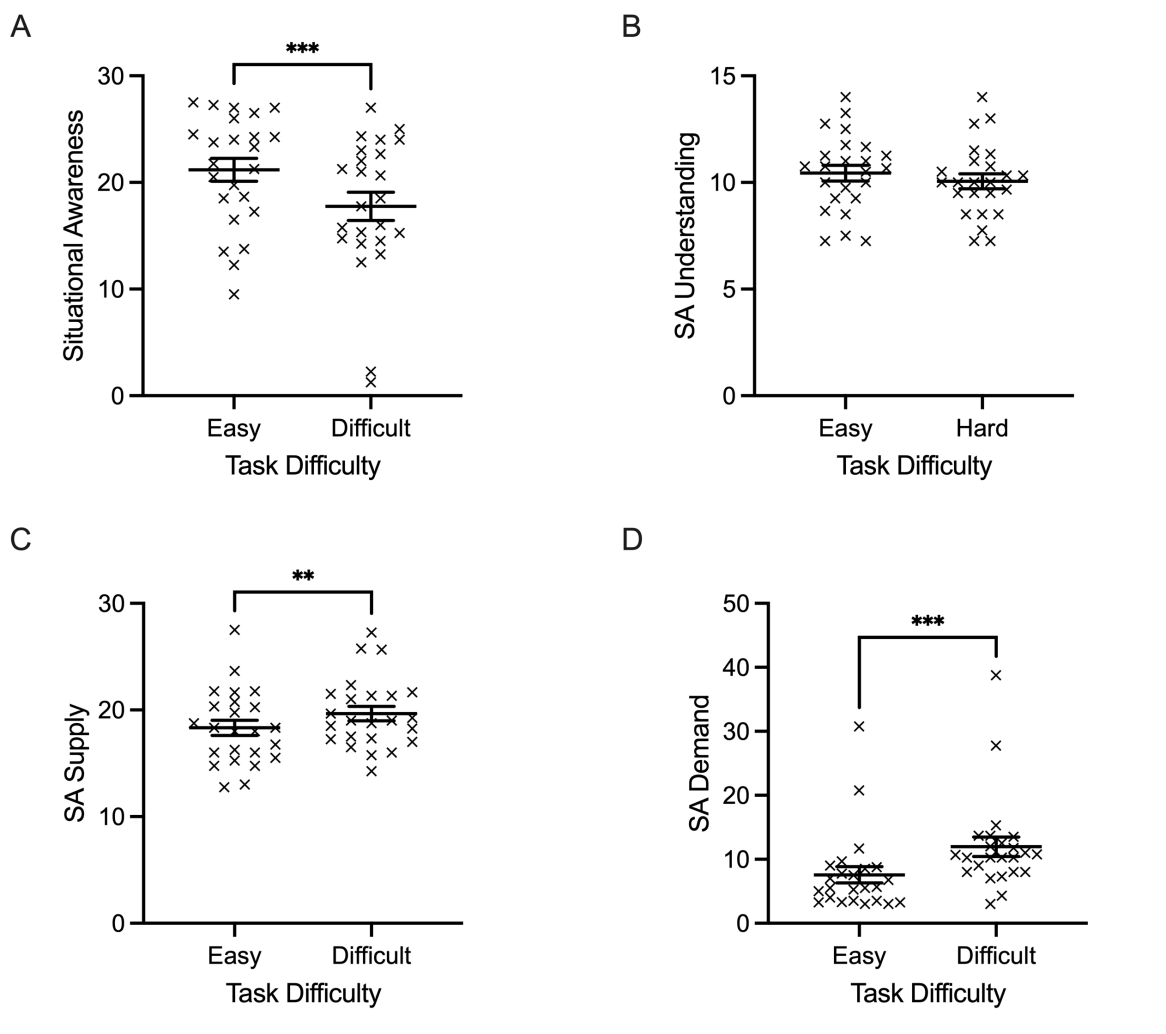
Individual data points and their respective group means for Situation Awareness (SA) Score (A), SA Understanding subcomponent score (B), SA Supply subcomponent score (C), and SA Demand subcomponent score (D) are demonstrated for easy and difficult conditions. Error bars represent SEM. *p ≤0.05, **p ≤0.01, ***p ≤0.001.

*Situation awareness.* Subjective scores from the SART questionnaire produced a general SA score, which demonstrated a main effect of task difficulty, *F*(1, 23)= 22.769, *p*<0.001, 𝜂_p_^2^= 0.497. Specifically, subjective SA scores were lower for difficult trials (X= 17.76, SD= 6.50) compared to easy trials (X= 21.19 SD= 5.23) (Figure 11A). A closer examination of the SART questionnaire subcomponents revealed that SA demand and SA supply also yielded a main effect of task difficulty, *F*(1, 23)= 57.280 and 13.931, *ps*≤0.001, 𝜂_p_^2^= 0.714 and .377, respectively. Figure 11 (c) & (d) shows how both SA demand and SA supply components increased in the difficult condition (SA demand: X= 11.96, SD= 7.35; SA supply: X= 19.66, SD= 3.29) compared to the easy condition (SA demand: X= 7.57, SD= 6.27 SA supply: X= 18.33, SD= 3.5). The SA understanding component did not reveal a main effect of task difficulty (p=0.140) (Figure 11B).

**Figure 12.**
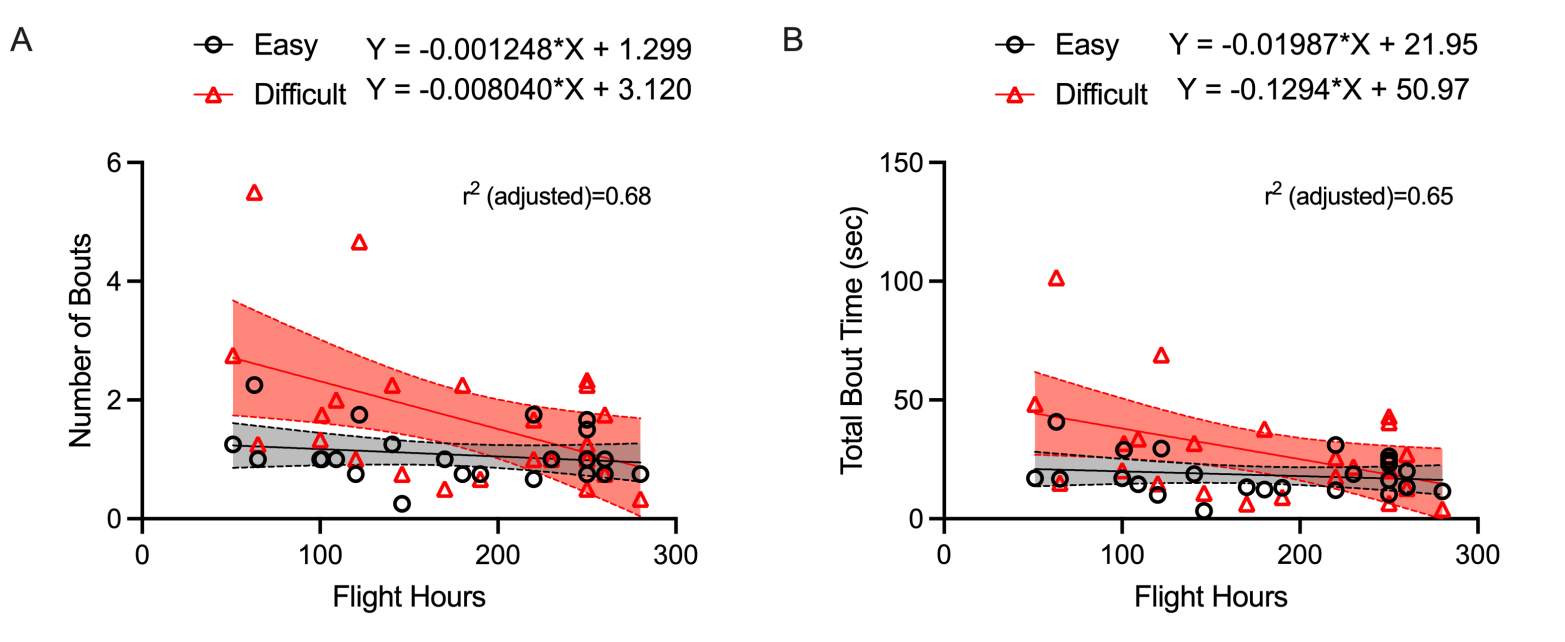
Scatter plots demonstrate the relationship between the number of cognitive tunneling bouts and pilot flight hours (A), and the total cognitive tunneling bout time (sec) and pilot flight hours (B). Black circles indicate easy condition participant averages, red triangles indicate difficult condition participant averages. Lines indicate the trend for all subjects (i.e., line of best fit). Highlighted bands around line of best fit indicate the 95% confidence interval.

*Regression Analyses.* A linear mixed model analysis was run in R Studios (version 4.3.1) to examine the relationship between the developed dynamic gaze metrics (i.e., number of cognitive tunneling bouts and total bout time) and pilot flight experience (i.e., flight hours) (Table 5). In the first model, we included the number of bouts as the dependent variable, task difficulty as a fixed effect, flight hours as a random effect, and the interaction between flight hours and task difficulty. There was a main effect of task difficulty, *F*(1, 46)*=*17.29, *p*<0.001, 𝜂_p_^2^=0.44, flight hours, *F*(1, 46)= 4.71, *p=* 0.041, 𝜂_p_^2^=0.18, and an interaction between flight hours and task difficulty, *F*(1, 22)=8.88, *p*=0.006, 𝜂_p_^2^=0.29 (Table 5). Specifically, the slopes were significantly different between the easy and difficult conditions when compared between the same group of pilots, with more cognitive tunneling bouts being observed in pilots with lower hours during the difficult condition (Figure 12A).

**Table:**
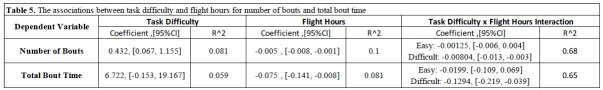


Note. Estimated marginal (EM) mean of linear trend characteristics for the number of bouts and total bout time models are provided including their respective coefficients (ß), 95% lower/upper confidence levels, and R-squared values.

In the second model, we included total bout time as the dependent variable, task difficulty as a fixed effect, flight hours as a random effect, and the interaction between flight hours and task difficulty (Table 5). Each subject was also included as a random effect. There was a main effect of task difficulty, *F*(1, 46)= 13.12, *p*=0.001, 𝜂_p_^2^=0.37, and an interaction between flight hours and task difficulty, *F*(1, 22)= 6.89, *p*=0.015, 𝜂_p_^2^=0.24 (Table 5). Specifically, the slopes were significantly different between the easy and difficult conditions when compared between the same group of pilots, with longer total gaze time being observed in pilots with lower hours during the difficult condition (Figure 12B). Note that regression analyses involving all other dependent variables did not reach significance.

## Discussion

Aviation accidents related to human error have been increasingly associated with poor pilot monitoring as it can negatively impact the degree to which operators receive adequate information to understand and predict the changing circumstances; thus, directly impact performance and SA (11; 37; 46; Stanton et al., 2017). As such, the current study provides one of the first accounts for how gaze behaviour changes simultaneously with flight performance and SA throughout the various stages of landing (i.e., downwind, base, final approach) during different task difficulty conditions to gain insight into low-time pilot monitoring patterns. Participants were asked to perform the landing task in high visibility, visual flight rules (VFR) conditions that included a task difficulty manipulation based on the absence (easy) or presence (difficult) of strong crosswinds. Several notable contributions emerged from this study. First, there was partial support of our initial hypothesis that gaze behaviour would reflect a greater need to allocate more time and attention toward fewer, task critical AOIs during the difficult condition. Specifically, a reduction in attention allocation toward fewer cockpit AOIs was coupled with an increase in cognitive tunneling behaviour during the difficult condition. Moreover, the allocation of attention across most AOIs was significantly impacted by flight phase. Second, an exploratory analysis that employed linear mixed model regressions found significant associations between dynamic gaze metrics and pilot flight hours. These contributions and their implications for integrating gaze behaviour analysis into pilot training and assessment are discussed below.

### Examining the effectiveness of the task difficulty manipulation

Landing performance data provided some evidence that task difficulty was effectively manipulated using varying weather conditions (i.e., no wind versus significant crosswind); however, some results were contrary to what was expected. Similar to previous work (5; 19), completion time was ~68 seconds (~39%) longer in the difficult condition compared to the easy condition. Although the difficult condition required more time to complete, task performance data demonstrated a speed-accuracy trade off in that other flight performance parameters were improved. This contradicts what was reported in previous work (5), where more difficult scenarios were associated with longer completion times and increased landing error. However, it is important to recall that the current study recruited a more homogenous pilot cohort with more flying experience; and thus, they might have been able to handle the task difficulty manipulations used in previous work with less experienced participants (5). For instance, the current study demonstrated that the difficult condition was also associated with a reduction in landing hardness (~30 fpm), airspeed RMSE and variability, as well as vertical speed RMSE and variability. This reduction in error and performance variability was specifically seen during the final approach stage of flight, which was shown to have the highest error and variability across all stages of landing (i.e., downwind, base, final approach). Notably, pilot performance during the easy condition was not necessarily bad. According to Transport Canada landing guidelines for PLL pilots, the observed landings were all considered successful and safe. Pilots simply performed better during difficult trials, likely because they were being more attentive to the task demands and performing the task with a higher degree of effort, particularly during final approach. Work by Stuhr and colleagues (49) further supports this assertion as they demonstrated that the impact of cognitive control processes on motor skill proficiency depends on performance variability. In other words, when performance variability is high (i.e., typical in novel or highly complex/difficult motor tasks such as the final approach phase of flight in this study), individuals are more likely to engage cognitive control processes to assist in the successful performance of the task at hand.

Another potential account for these results could be that the final approach phase of flight was the only phase of flight that had 22 knots of headwind; a component that was not present in previous work (5). Headwind is notoriously known to make it easier for pilots to control the aircraft as it provides additional lift to the aircraft and at lower speeds (24). This in turn would be associated with improved aircraft control and landing performance (i.e., reduced variability and deviation from pre-set parameters) (Figures 4-6). Since both accounts can explain the reported results, more research is required to fully understand how the performance parameters are being modulated by the task difficulty manipulation used in the current study. Specifically, future work could examine an alternative task difficulty manipulation that doesn’t involve the introduction of winds, introduces head winds during the final approach but no cross wind to the “easy” trial, or significantly reduces the headwind component encountered during the final approach phase of the landing scenario.

From the performance measures alone, it could be argued that the task difficulty manipulation did not necessarily increase the difficulty of the task itself, but rather made it more engaging. However, the additional cognitive resources devoted to maintaining/improving task performance were likely reallocated from other task-related aspects of flight performance, such as SA or changes in information processing (demonstrated through changes in gaze behaviour). Indeed, seminal research has shown that SA plays a critical role in pilot performance as it involves the degree to which operators *receive* (i.e., perceive and process) adequate information to *understand* the changing conditions in their environment, and *project* the impact on future circumstances (21). This is a crucial aspect of aircraft control to consider, because even though flight performance may not be overtly different across various flight scenarios (i.e., changes in wind conditions), a pilot’s ability to receive incoming information, comprehend it, and project its impact on future circumstances may still be impaired, which will affect their ability to problem solve and make informed decisions should an unexpected event (i.e., in-flight emergency) occur. The current study demonstrated that subjective SA, as indicated through the SART, decreased by ~8% in the difficult condition relative to the easy condition (Figure 11). An in-depth assessment of the SART SA sub-components revealed that attentional supply (i.e., arousal, spare mental capacity, level of concentration, division of attention) increased by approximately 5% (Figure 11), which supports the earlier claim that participants were being more attentive to task demands and exerted more effort during the difficult condition. However, this was substantially overshadowed by the ~21% increase in the attentional demand sub-component (i.e., how instable, variable, and complex was the situation) (Figure 11).

In light of the reduction in aircraft control error and variability during difficult trials, the SA results are interesting in the sense that improved aircraft control was hypothesized to be associated with an increase in SA, whereas a reduction in SA was assumed to be associated with poorer aircraft control. Three alternative explanations are proposed to account for the reported findings where pilot performance improvements were seen in parallel with a reduction in SA. First, an argument could be made that subjective SA as measured through the SART may be more reflective of participant confidence level and not necessarily a true representation of SA (20; 44). As such, the reduced SART scores may indicate that participants were less confident about their performance during the difficult condition as they had to devote more attentional resources toward the task relative to the easy condition. A second alternative explanation could be that if pilots were concentrating more on aircraft control during the difficult condition, perhaps this was at the cost of no longer attending to other things going on around them; thus, resulting in a reduction in SA. A third explanation could also argue for the existence of a ‘buffer’ effect that may be associated with pilot proficiency. In other words, a reduction in SA may be compensated with greater pilot proficiency when managing increased task demands, as they will have a lower chance of exceeding the pilots’ cognitive resources (12; 13; 16). Since the current study recruited pilots who were already licensed, and thus experienced in flying the simulated aircraft under the tested flight conditions (i.e., little-no winds and high crosswind conditions), it is likely that they had the capacity to effectively devote additional cognitive resources to further support task performance in the presence of reduced SA during the difficult landing condition. The gaze results (discussed below) suggest that the second and third explanations may be plausible accounts for the reported findings as there is evidence of a broad reduction in the scanning of environmental stimuli, but there is also data suggesting that some changes in gaze behaviour work to compensate for increases in task difficulty that might help prevent reductions in flight performance. Still, these assertions should be further tested in future research that significantly challenge the recruited pilot pool and examines how different gaze patterns align with how pilots manipulate aircraft control inputs (i.e., throttle, pitch, roll inputs).

In addition to the reported SA findings, several gaze variables were significantly modulated by increases in task difficulty. For instance, blink rate was reduced (Figure 8), the allocation of attention was selectively increased toward the front window while it decreased across a number of other less-relevant AOIs (Figure 7), and the frequency and total duration of cognitive tunneling gaze behaviours increased when task difficulty increased (Figure 10). Note that the dwell time findings reported here differ from earlier work (5), which reported an increase in front window dwell time during the difficult condition compared to the easy condition. The previous study suggested that this change in gaze behaviour reflected the need to allocate more attention toward fewer task critical AOIs; of which, the front window was a prime source for monitoring and extracting necessary information required to land during challenging wind conditions (5; 8; 18; 27; 30; 35; 43). In partial support for this expected finding, a comprehensive analysis of dwell time changes during each stage of flight demonstrated a reduction in attention toward the left window and airspeed indicators, but it failed to demonstrate a significant increase in dwell time toward the front window. This was further examined by aggregating the gaze data for a broader analysis of dwell time patterns across the AOIs during difficult and easy scenarios without the segregation of data by flight phase. This additional analysis confirmed what was reported in earlier work showing a reduction in a number of AOIs (i.e., airspeed, attitude, altimeter, left window) and an associated increase in front window dwell time (~10%). Accordingly, we suggest that analysis of gaze data across various phases of flight is appropriate to assess information processing changes associated with the different sub-goals linked to each stage (17). However, the segregation of time-normalized dwell time data into stages of landing negatively impacts our ability to draw conclusions about how specific patterns of attention allocation across numerous AOIs- which have variable time courses- are impacted by task difficulty. It is also important to note that this limitation may be a consequence of a reduction in power for this specific analysis. Previous work examined task difficulty changes across all AOIs but did not further segregate the data by landing stage (5). As such, it may also be the case where more participants would have been required to properly replicate previous work with the additional phase of flight variable. Nevertheless, these shifts in gaze behaviour are proposed to reflect a necessary shift in top-down attentional controls imposed on visual scanning during challenging task demands to help focus attention to the appropriate object at the appropriate time (5; 6; 10; 12; 23; 47). Moreover, the blink rate findings corroborate the suggested relationship that blink rate depression is associated with an increase in task difficulty as the current findings demonstrated the lowest blink rate to coincide with the most challenging phase of landing (i.e., final approach) (26; 40; 64).

An interesting gaze outcome that was contrary to what was hypothesized was the significant increase in SGE and GTE during the difficult trials, particularly during the final approach stage of landing. Previous work demonstrated that increases in task difficulty were associated with a reduction in SGE and GTE (5). Several reasons may help to explain the divergent results. First, it is important to remember that the cohort in the previous study consisted of ~56% ab-initio pilots with little to no flight experience while the current study exclusively enrolled licensed pilots. Conceivably, flight experience has a significant effect on gaze behaviour. For instance, licensed pilots may be more aware and capable of increasing their gaze dispersion and sequence complexity to gather more information in a more efficient manner to aid in the management of crosswind conditions (i.e., lateral slip/crabbing methods to enhance aircraft control) (12; 13; 24). Second, it is possible that the pilots recruited in the current study were not challenged to the same extent as the participants recruited in previous work (5). As such, it could be hypothesized that the SGE and GTE would similarly decrease in response to a significantly more difficult condition if the current study provided a greater challenge. In this case, the hypothetical SGE and GTE reductions may also reflect a shift in top-down attentional control mechanisms to selectively allocate visual attention and focus visual scanning to highly critical AOIs when task difficulty is high (5). Future work should include a significantly more difficult task (e.g., dual-task paradigm) to stress the cognitive control resources of pilots during a simulated flight. Since the reported increase in gaze scanning distribution and sequence complexity (Figure 9) were seen in parallel with a reduction in aircraft control variability (Figure 5 & 6) during the final stage of flight for the difficult relative to the easy condition these particular gaze data are suggested to reflect a compensatory mechanism to support task performance (31).

### Flight phase parameters and experience influence information processing

The findings presented clearly demonstrate that all traditional gaze metrics (i.e., dwell time percentage, dwell rate, and average dwell duration across AOIs) vary significantly between flight phases, which most likely reflects the differing task sub-goals specific to each stage of flight (5; 7; Dehais et al., 2021; 18; 26; 27; 30; 43; 54). For instance, during the downwind flight phase participants are normally completing their landing checklists and flows to configure the plane for landing (24). This was manifested in the downwind gaze patterns which had the highest dwell times for the front window, altimeter, power, and airspeed indicators, which are the various gauges that require attention when configuring the plane to land (i.e., continue flying straight ahead [antiparallel to runway heading], reduce power to 15,000 rpm, maintain circuit altitude, and ensure airspeed is configured for this landing stage [100kts]). Moreover, further exploration of non-AOI data demonstrated that the greatest amount of time devoted to looking at the landing checklist and other configuration regions not included in the 10 pre-defined AOIs was during the downwind phase of flight (16%). Although this was not included for analysis in the current study (<5% of total dwell time), it provides additional support for how gaze behaviour supports flight phase-specific task goals. The base leg of flight was associated with the highest dwell times for the airspeed, attitude, and the left and front window AOIs. This was tied to the fact that pilots were required to complete the turn-to-base and turn-to final approaches during this leg of flight while maintaining the recommended flight speeds (24). As such pilots frequently monitored aircraft roll via the attitude gauge, landmarks that helped determine when to begin and end the turn (i.e., runway visual via the left window), and the airspeed gauge to adjust airspeed as needed in between stage transitions (i.e., downwind= 100 kts, base= 70 kts, final= 65 kts). Last, the main goals of the final approach stage of landing were to maintain the glide slope, center alignment with the runway, and transition the aircraft from a normal approach attitude to a landing attitude (i.e., flare) (24). Visual cues that help monitor each of these goals are predominantly located through the front window and serve as the main reason why the allocation of attention to this AOI is greatest during the final approach (5; Dehais et al., 2021; 18; 26; 27; 30; 43). The airspeed gauge was also a critical AOI as final approach speed is important for maintaining optimal glide slope and preventing an engine stall. These findings were supported by similar, though less robust, trends in the dwell rate and average dwell duration data.

In line with the traditional gaze findings, more computationally complex measures of gaze behaviour including SGE and GTE demonstrated significant reductions from the downwind/base stages of landing to the final approach stage of landing (Figure 9). This further supports the claim that gaze behaviour changes are contingent on flight phase specific sub-goals. For example, higher SGE and GTE suggest increased dispersion and more gaze shifts across the AOIs that require continual monitoring to configure the flight during the downwind and base stages compared to the final approach stage of flight; wherein the focus of attention routinely becomes restricted to 2-3 AOIs. With respect to other dynamic gaze measures of information processing, the analysis of the moment-to-moment changes in gaze fixation and dispersion provided insight into broader changes pilot monitoring and cognitive tunneling (i.e., prolonged outside gaze fixation) behaviours over time which were influenced by the task difficulty manipulations, but more so dictated by flight experience (i.e., flying hours). In line with previous work (5), the current study demonstrated an increase in cognitive tunneling bouts and total cognitive tunneling bout time in the difficult condition compared to the easy condition. In other words, the difficult condition was associated with more frequent periods of time where pilots neglected to scan their cockpit. Although this could have resulted in a tunneling of attention toward any other AOI, what was observed in previous studies (1; 2; 5; Ayala et al., 2024; 59) alongside current work was a fixation of attention outside. This is a critical aspect of gaze behaviour to examine as a lack of scanning during phase(s) of flight that require continuous monitoring of flight parameters (i.e., landing) and aircraft state via gauges inside the cockpit can result in disastrous consequences should an unexpected/hazardous event occur. This phenomenon was similarly reported in driving simulation studies that found that the higher proportion of dwell time allocated to the center of the road and a reduction in gaze dispersion were associated with driver distraction, reduced hazardous event detection, and increased driver cognitive load (41; 58; 60; 61). The increased cognitive tunneling findings seemingly contradict the increase in final approach SGE and GTE during difficult trials. However, it is important to remember that the dynamic bout analysis is examining duration-based cognitive tunneling tendencies over time while time-averaged entropy measures only consider the frequency and sequence of AOIs (14; 45; 47). Moreover, the cognitive tunneling bout analysis provides a marker of poor monitoring behaviour that is independent of flight stage and instead considers the duration of time that gaze is diverted from cockpit gauge monitoring across the entire trial whereas the SGE/GTE findings in question are demonstrated only for the final approach phase of flight (5; 59; 64). In fact, when SGE and GTE are averaged across the entire trial, there is no effect of task difficulty; a finding that suggests that although fixation dispersion and gaze sequence complexity don’t change as a function of task difficulty in the recruited participant pool, the cognitive tunneling behaviour (i.e., cockpit monitoring behaviour) still remains an informative measure of changes in information processing. Moreover, the current study makes an important contribution by further demonstrating how pilot experience affects cognitive tunneling behaviour. The linear mixed model regressions demonstrated that cognitive tunneling events account for up to 68% of variance in pilot flight hours; a finding that provides evidence for the claim that dynamic cognitive tunneling bout analysis is a robust measure of pilot monitoring behaviours that change with experience. This is significant because it provides the first gaze-base measure that can identify differences in pilot proficiency within a tightly defined recruitment pool (i.e., flight hours range: 51-280hrs). Furthermore, the bout regression analysis provides additional support for the extent to which low time pilots need to be challenged before one can observe meaningful changes in gaze behaviour as a function of flight hours. This is demonstrated by the task difficulty by cognitive tunneling (number and total time) interaction suggesting the relationship for the currently defined flight hour range is present, but only in the more difficult condition when the capabilities of pilots with fewer flying hours are being challenged.

Notably, the implementation of a number of gaze metrics examined in the current study (i.e., dwell time phase plots, SGE/GTE, and cognitive tunneling) into pilot training and assessment protocols could provide easily interpretable metrics that are objectively related to pilot proficiency levels, and identify instances where pilots demonstrate deviations from optimal monitoring behaviours. In example, dwell time plots may be used to assess where pilots are allocating their attention and if it aligns with task-relevant sources of information that require visual processing, SGE/GTE plots can aid in identifying pilots who are deviating from pre-determined task norm values, and cognitive tunneling measures can be used to assess poor monitoring behaviours that directly align with International Air Transport Association (IATA) competency assessment questions about “How Many” and “How Often” (36) these behaviours occur. These capabilities further enhance the extent to which gaze metrics, such as those described here, can be used as an assessment and training tool.

### Limitations and future directions

The current study provides several novel insights into the way task difficulty and flight phase parameters impact task performance, gaze behaviour and SA. However, the current results are constrained by at least four methodological limitations. First, the tight pilot recruitment pool limited the extent to which the regression model analysis could relate flight experience (i.e., flying hours) to dynamic gaze measures (i.e., number of cognitive tunneling bouts and total cognitive tunneling bout time). Future studies seeking to gain a better understanding about the utility of the novel dynamic gaze measures (5) in characterizing pilot flight hours or SA should include a larger range of pilot experience backgrounds that extend from the early ab-initio level to the instructor pilot level. Second, the extent to which other relationships could be thoroughly assessed was also undermined by the small participant pool. For example, the ability to examine the relationship between dynamic gaze behaviours and performance or SA was limited by the fact the models demonstrated a lack of power. As such, no conclusion could be made about the utility in using cognitive tunneling bout analysis to ascertain pilot SA or task performance. This could be improved in future work by increasing the number of trials each participant completes, or by conducting the experiment in Instrument Flight Rules (IFR) conditions where dynamic gaze behaviour and/or SA would be stressed. A third related limitation was that there was no singular measure of landing performance that could be used to effectively determine relationships between flight performance and SA or dynamic gaze metrics. As such, future work should involve instructor pilots that are familiar with the assessment of landing performance in single engine aircrafts to provide evaluations for these analyses. This can be further supported using the NASA-TLX as it would provide more support for subjective ratings of task difficulty/load compared to the SART. Lastly, our conclusions about the task performance and entropy changes seen in the difficult condition being attributed to an increase in attention and exerted effort is a fascinating finding; and one that should be further examined when cognitive resources are exceeded by task demands. In other words, both accounts for the current findings could be strengthened with additional testing to determine how task performance and gaze dispersion/sequence complexity are impacted when cognitive load is significantly higher. For instance, introducing a secondary task (i.e., dual task paradigm) to further challenge cognitive resources may reduce the extent to which additional cognitive control resources are able to modulate gaze behaviours and assist in task performance.

### Conclusion

This work highlighted the performance, SA, and gaze behaviour differences in low-time pilots when completing a simulated landing scenario in VFR conditions with and without the presence of strong crosswinds. Traditional gaze metrics (i.e., dwell time, rate, duration, blink rate) and entropy-based metrics all provided meaningful insight about the extent to which task demands and information processing change across the different phases of flight (i.e., downwind, base, and final approach) and task difficulty. Our results suggest the changes in gaze behaviour compensated for the increased task demands and minimized the impact on task performance, particularly during the final approach stage of landing. Lastly, the cognitive tunneling analysis remains a robust measure of task difficulty and, more importantly, pilot experience (i.e., flight hours), which accounted for up to 68% of variance in the moment-to-moment analysis of pilot monitoring behaviour (i.e., number of cognitive tunneling bouts and total bout time). In conclusion, a number of traditional and advanced gaze metrics provided critical insight into how gaze behaviour, and thus, information processing, is altered by task difficulty and task goals. However, more work is needed to validate its utility in being able to characterize pilot proficiency.

### Ethics and Conflict of Interest

The author(s) declare(s) that the contents of the article are in agreement with the ethics described in http://biblio.unibe.ch/portale/elibrary/BOP/jemr/ethics.html and that there is no conflict of interest regarding the publication of this paper.

### Acknowledgements

This research was supported in part by grant 00753 from the New Frontiers in Research Fund.

We wish to thank Diako Mardanbegi of AdHawk Microsystems for providing the areas of interest gaze mapping script used in this article.
